# The Mediating Role of Parenting Stress in the Relations Between Parental Emotion Regulation and Parenting Behaviors in Chinese Families of Children with Autism Spectrum Disorders: A Dyadic Analysis

**DOI:** 10.1007/s10803-019-04103-z

**Published:** 2019-06-13

**Authors:** Xiaoyi Hu, Zhuo Rachel Han, Liu Bai, Mengyu M. Gao

**Affiliations:** 1 Department of Special Education, Education Research Center for Children with ASD, Faculty of Education, Beijing Normal University, Rm 408, YingDong Building, Xin Jie Kou Wai Da Jie #19, Beijing, China; 2 Beijing Key Laboratory of Applied Experimental Psychology, National Demonstration Center for Experimental Psychology Education, Faculty of Psychology, Beijing Normal University, Rm 1312 Hou Zhu Lou Xin Jie Kou Wai Da Jie #19, Beijing, China; 3 Department of Human Development and Family Studies, College of Health and Human Development, The Pennsylvania State University, University Park, USA; 4 Department of Psychology, University of Notre Dame, Notre Dame, USA

**Keywords:** Autism spectrum disorders, Dyadic analysis, Emotion regulation, Parenting stress, Parenting behaviors

## Abstract

Little is known regarding the dynamic interactions between fathers and mothers in families of children with autism spectrum disorder (ASD) during the parenting process. This study used an actor–partner interdependence mediation (APIMeM) model to investigate the intrapersonal and interpersonal effects of emotion dysregulation and parenting stress on parenting behaviors among 211 pairs (total N = 422) of Chinese parents of children with ASD. The results indicated that for both fathers and mothers, there were significant indirect actor effects of parental emotion dysregulation on parents’ own parenting behaviors through their own parenting stress. However, no significant direct or indirect partner effect was found in the analyses. These findings suggest that the emotional parenting dynamics occurred on the individual rather than the dyadic level in these families.

## Introduction

Raising a child with autism spectrum disorder (ASD) can exert tremendous stress on parents (Davis and Carter 2008) given that the core features of ASD include impaired social communication, restricted interests, and repetitive behaviors (American Psychiatric Association 2013). Numerous studies have demonstrated that these families experience more stress than families with typically developing children or children with other special needs (e.g., Brei et al. 2015; Falk et al. 2014). Although these findings are unsurprising given the chronic nature of the challenging behaviors associated with ASD, they emphasize the importance of identifying factors that may contribute to the stress that parents perceive and the degree to which such stress might affect their actual parenting behaviors (Hayes and Watson 2013; Keenan et al. 2017).

However, the literature has not yet fully addressed how parents’ own abilities (e.g., emotion regulation) might influence their parenting stress, nor has the research ventured far into more direct links between parenting stress and parenting behaviors in families of children with ASD, especially considering these family processes in a dyadic manner. This lack of literature might be accounted by the common practice in ASD research of avoiding potentially negative depictions of parents. This avoidance may be due to the historically discarded refrigerator mother hypothesis, which blamed parents, especially mothers, as the primary cause of their children’s ASD (Silverman 2012). Nonetheless, more research delineating the parenting processes in families of children with ASD can help provide more targeted support for parents with various psychological characteristics. Indeed, understanding what contributes to perceived parenting stress and how such stress further shapes the parenting behaviors of fathers and mothers raising children with ASD will inform targeted interventions to support parental well-being and facilitate family functioning (Hayes and Watson 2013; Zaidman-Zait et al. 2018). Such research is especially critical in populous countries such as China, where it is believed that at least 1.3 million families are affected by ASD (Huang et al. 2013) and where ASD has become a nationwide public health concern (Jin et al. 2018).

### Parenting Stress in Families of Children with ASD

Parenting stress refers to an aversive psychological reaction to the demands associated with being a parent (Deater-Deckard 1998). Elevated levels of stress associated with parenting may have serious negative effects on parents’ own parenting behaviors and on other family members’ outcomes. Abidin (1990) proposed a stress model within the parenting context, in which parenting stress is created by a mismatch between the perceived demands of parenting and the available social and personal resources to address these demands. Taking care of a child with ASD means constantly dealing with the challenges of comorbid physical and psychological symptoms, high costs of treatment and limited work time, as well as the associated stigma and self-blame among Chinese parents (Liang 2018). Thus, it is not surprising that parents of children with ASD might tend to perceive elevated levels of parenting stress.

### Emotion Regulation and Parenting Stress

Despite ample evidence demonstrating the generally heightened levels of parenting stress in families of children with ASD (e.g., Dardas and Ahmad 2014), previous research has suggested that parents vary substantially in their ability to cope with the challenges associated with their children’s autism (e.g., Benson 2010, 2014). This interindividual difference could be due to the difference in their perceived parenting stress as a result of their own levels of emotion regulation, given that emotional coping abilities are important components of personal resources for dealing with environmental stressors (Deater-Deckard et al. 2016). Indeed, parenting per se is an emotional experience. However, very little research has explored the emotional aspects of parenting within families of children with ASD or how these emotional aspects might affect their perceived parenting stress. Even less research has considered both fathers’ and mothers’ effects on these links in a dyadic manner.

In terms of emotional coping abilities in parenting, parental emotion regulation might be among the most important indicators. Emotion regulation has been widely conceptualized as the internal and external processes involved in initiating, maintaining and modulating the occurrence, intensity, and expression of emotions in order to accomplish one’s goals (e.g., Gross 2011; Thompson 1995). From the functionalist perspective, emotion regulation or dysregulation is considered in terms of the social context of that emotion; only when emotional responses are not appropriate for a certain context or interfere with an individual’s behavior and psychological functioning do we consider such behavior as a sign of dysregulation (Cole and Hall 2008).

Many have argued that culture might also shape emotional competence (including emotion regulation) and therefore influence an individual’s parenting outcomes (e.g., Friedlmeier et al. 2011). Therefore, more research is needed to better understand emotion regulation beyond the Euro-centric models of emotional competence (Suveg et al. 2014). Hofstede (1980) initially proposed the individualism–collectivism dimension to help describe the primary differences among various cultures. In terms of emotion regulation within different cultural contexts, it seems that individualistic societies (e.g., the US) value independence and focus on the self, thus encouraging the expression of ego-focused emotions (e.g., anger). In contrast, collectivistic cultures (e.g., China) emphasize interdependence and group harmony, thus encouraging the suppression of ego-focused emotions (Mesquita and Frijda 1992). These differing attitudes toward when certain emotions should be downregulated or upregulated in collectivistic versus individualistic cultures require us to consider cultural contexts when examining emotion regulation. Given that the relationship between emotional competence and parenting outcomes are comparatively understudied in collectivistic culture, the current investigation aimed to address this issue in a collectivistic society (i.e., China).

Parental emotion regulation is believed to play an important role in child development given that parents must maintain a regulated state to facilitate regulation in their relationship with children when providing care (Bariola et al. 2011). For example, parental emotion regulation within the family context is of particular importance because the family is the primary context in which children learn about the rules for displaying emotions and gain an understanding of others’ emotions (Halberstadt et al. 1995). Previous research in families with typically developing children has also described the links between parental emotion regulation and parenting stress. First, it has been documented that difficulties with emotion regulation are related to stress in general from both psychological and physiological perspectives (Wang and Saudino 2011). Parents with poor emotion regulation tend to perceive parenting responsibilities as more stressful than those with better emotion regulation (Deater-Deckard et al. 2016). This might be particularly true for parents of children with ASD as the stress associated with parenting these children is already overwhelming. Second, many have argued that stress could be regarded as “a subset of emotion” (e.g., Lazarus 1993). Considering that parenting stress involves negative feelings toward both one’s partner and children (Deater-Deckard 1998), it is likely that emotion regulation plays a key role in dealing with the stress associated with parenting.

In addition to the effect of parental emotion regulation on a parent’s own perceived stress when parenting the child, there might be some dynamic interactions between fathers’ and mothers’ emotion regulation and parenting stress. According to the crossover hypothesis from family systems theory, emotions and behaviors from one family member might influence other members in the same family (Nelson et al. 2009; Cox and Paley 1997, 2003). Thus, the negative effect of one parent’s failure to cope with his/her own emotions might transfer to the relationship between the other parent and the child. For example, the frequent negative emotional collapse of one parent might lead the other parent to feel more stressful when taking care of their child with ASD. Based on these theoretical foundations, it is optimal to consider the dynamic exchanges between fathers’ and mothers’ emotion regulation and parenting stress when examining their co-parenting of their developmentally challenged children. Indeed, there is an emerging trend in the literature to perform dyadic examinations in families of children with ASD to better account for the interaction between mothers and fathers. For example, Ekas et al. (2015) found that in parents of children with ASD, mothers’ and fathers’ use of emotional support from their partners highly predicted their relationship satisfaction. In line with this finding, García-López et al. (2016) also found that the relationship satisfaction of mothers and fathers of children with ASD mediated the association between their supportive dyadic coping strategies and their own and their partner’s parental adaptation.

### Emotion Regulation, Parenting Stress, and Parenting Behaviors

In addition to the potential links between parental emotion regulation and parenting stress, the elevated levels of stress associated with parenting may also have serious negative effects on each parent’s parenting behaviors. The parenting literature on ASD has shown an emerging trend that during the early stages of child rearing, the parenting behaviors of parents of children with ASD do not seem to be significantly different from the parenting behaviors of parents of typically developing children, parents of children with intellectual disabilities, and parents of children with language disorder (e.g., Rutgers et al. 2007). However, among parents of youths with ASD aged 7 to 17 years old, more negative parenting (e.g., physical control, harsh parenting) and less positive parenting (e.g., parental warmth and sensitivity) have been found (Chang et al. 2018; Riany et al. 2017). Although these studies are from an Asian population that was culture-specific, one reason for the more negative parenting might be that the challenging behaviors of youth with ASD are more visible and therefore have a more explicit effect on parenting.

One way that parenting can be measured is through parents’ general patterns of childrearing, which characterize their typical responses to a broad range of contexts and situations (Coplan et al. 2002; Darling and Steinberg 1993). Such behaviors can be summarized as parenting behaviors that promote or disrupt their “optimal parental bonding” with their children and can be broadly categorized into “care/affection” and “overprotection/control”. Parents who demonstrate more care/affection tend to be more positive during their interactions with their children and exhibit more emotional warmth, closeness, and empathy toward their children, whereas overprotective parents tend to control every aspect of their children’s lives and encourage dependence, intrusion, and control of their children’s behaviors (Parker et al. 1979; Rubin et al. 2002). In a study of Taiwanese children with ASD, parents were found to be less affectionate and more overprotective than parents of typically developing children (Gau et al. 2010). Generally, parental care/affection has been consistently associated with positive outcomes, such as better emotion regulation skills and fewer behavioral problems, whereas parental overprotection has been consistently associated with poor adjustment among children in at-risk samples (e.g., Gere et al. 2012).

Previous studies have revealed that Chinese mothers of children with ASD reported providing lower levels of maternal care and higher levels of maternal overprotection toward their children (e.g., Chang et al. 2018). These findings are in contrast with those found among their Western counterparts (e.g., Ventola et al. 2017). Many researchers have attributed such patterns to Chinese parents’ concern about their children with ASD’s lack of safety awareness and ability to cope with stress. However, little has been done to explore how parental factors might explain such findings.

The affective structure of parenting theory validates the fact that parents’ emotional states regulate parenting behaviors and that dysregulated emotions are associated with fewer adaptive parenting behaviors (Dix et al. 2004). We further argue that parenting stress might mediate the relations between parents’ emotional abilities and parenting behaviors. That is, parents’ own emotional difficulties might lead to more maladaptive and less adaptive parenting because such difficulties limit the emotional resources available for dealing with chronic parenting stress (such as the stress associated with the long-standing role as primary caregivers of a child with ASD). Such associations might further correlate with a wide array of risks for maladaptive parenting. Indeed, given that perceived parenting stress varies greatly among parents, emotion regulation abilities might set the tone for how much parenting-related stress an individual subjectively perceives. The more stress one believes she or he has experienced, the less likely they are to provide optimal parenting to their children, especially those with ASD (Deater-Deckard 1998). To our surprise, very limited research has fully explored this important pathway in families of children with ASD or considered both fathers and mothers in investigations of parenting emotions, stress, and behaviors, despite the important role of parents’ emotional well-being in parenting (Dix 1991; Adam et al. 2004).

### Father’s Role and Dyadic Approach

Although there is evidence suggesting that fathers play an important role in family functioning, a paucity of family research has examined the experiences of fathers compared to those of mothers (e.g., Tomeny 2017; Zaidman-Zait et al. 2018). However, fathers have an irreplaceable role in parenting, and there are differences between fathers and mothers in terms of the level of parenting they provide (Bai and Han 2016; Lee et al. 2011). This might be especially true in families of children with ASD as autism is currently considered the developmental disability with the greatest impacts on the ordinary parenting role of fathers; having a child with autism leaves fathers significantly distressed, helpless, and vulnerable to many psychosocial problems (Lyons et al. 2010). However, the majority of research on fathers of children with ASD has only focused on fathers’ parenting stress and how they cope with such stress (e.g., Argumedes et al. 2018; Dardas and Ahmad 2014); fathers’ general emotional functioning and parenting behaviors as well as how those factors are associated with fathers’ parenting stress remain less clear.

Moreover, the crossover hypothesis of family systems theory emphasizes the interactions between fathers and mothers (Cox and Paley 1997; Erel and Burman 1995), and the particularly important role of co-parenting relationships within families of children with ASD has also been highlighted (Thullen and Bonsall 2017). Indeed, couples share the responsibility for childrearing, and each member of the couple receives support from the other (Brobst et al. 2009; Feinberg 2003). Spousal support has been associated with parenting stress (Abidin and Brunner 1995) and parenting outcomes (e.g., Allen and Hawkins 1999). Because individuals who have difficulties with emotion regulation may provide little emotional support and may potentially cause more marital conflicts (Bloch et al. 2014), we proposed that co-parenting a child with ASD with a spouse who has poor emotion regulation abilities is likely to make the parenting process more stressful, which might further impede the individual’s abilities to provide adaptive parenting. Therefore, we examined the emotion regulation, parenting stress, and parenting behaviors of both mothers and fathers, considering the family as the unit of analysis and testing the parents’ interactions.

### The Current Study

Based on these theoretical and empirical foundations, this study is among the first to investigate whether the parenting stress of Chinese fathers and mothers of children with ASD mediates the relationship between their own and their partners’ emotion regulation and parenting behaviors. Given that people in dyadic relationships (e.g., fathers and mothers) often influence one another’s thoughts, emotions, and behaviors, it is optimal to use an actor–partner interdependence model in the study of dyadic relations (Kenny et al. 2006). The specific research questions and hypotheses are as follows: (1) Is parental emotion regulation associated with both the actor’s and his/her partner’s stress? It is expected that parents who have more difficulties with emotion regulation will report experiencing more parenting stress themselves and that their spouses would also report more parenting stress. (2) Is parenting stress associated with both the actors’ and their partners’ parenting behaviors? It is expected that parents who report more parenting stress will engage in more negative parenting (overprotection/control) and less positive parenting (care/affection) and that their partners would demonstrate the same parenting patterns. (3) Does parental emotion regulation have an indirect effect on parenting behaviors through parenting stress? It is expected that parents’ emotion regulation is related to their parenting behaviors through their own and their partner’s parenting stress. That is, parents who have more difficulties with emotion regulation tend to report more parenting stress and subsequently report more negative and less positive parenting. Additionally, parents who have more difficulties with emotion regulation will have partners who report more parenting stress, which in turn will be linked with more negative and less positive parenting by themselves and their partners.

## Method

### Participants

A total of 226 families with children attending special education schools from 15 provinces in mainland China participated voluntarily. All the couples were parents (i.e., mothers and fathers from the same family) of children aged 7–12 years (*M* age = 10.35, *SD *= 2.63) with a clinical record of DSM-IV-TR diagnosis of ASD from qualified psychiatrists. The mean age at ASD diagnosis was 4.97 years (*SD* = 1.38), and the mean time elapsed since diagnosis was 5.37 years (*SD* = 2.66). Couples who failed to respond to more than 90% of the questionnaire items and those with children aged outside of this range (8 reported children younger than 6 years of age, and one reported a child aged older than 21 years) were excluded from analysis.

The final sample consisted of 211 couples (total *N* = 422, including 175 pairs of parents of boys and 35 of girls and one pair of parents of a child whose gender was not specified) with information reported by both the fathers (*M *= 41.68, *SD *= 5.65) and mothers (*M *= 39.20, *SD *= 4.64). A total of 210 mothers and 204 fathers identified themselves as biological parents. Regarding locations, 37% of the families (*N* = 77) were from rural areas (i.e., areas with less than 10,000 residents), and 63% of the families (*N* = 134) were from urban areas (i.e., areas with more than 10,000 residents). 56.4% of the families reported a yearly household income lower than 72,000 Chinese yuan (i.e., the low-income level); 19.9% of the families reported a yearly household income of 72,000–120,000 Chinese yuan (i.e., the low middle-income level); 11.8% of the families reported a yearly household income of 120,000–240,000 Chinese yuan (i.e., the upper middle-income level); and 7.1% reported a yearly household income greater than 240,000 Chinese yuan (i.e., the high-income level). According to the 2017 China national family yearly income standards (China Bureau of Statistics 2017), the yearly household income of the sample matched the national level (i.e., 62% of families at the low-income level, 21.9% of families at the low middle-income level, 8% of families at the upper-middle class level, and 8.7% of families at the high-income level). Regarding educational background, most of the mothers (46.4%) and fathers (51%) had received at least a college degree, 28.9% of the mothers and 24.8% of the fathers had a high school diploma, and the remaining parents had middle school or elementary education. All parents were currently married.

### Procedure

The parents received an invitation letter from their children’s teacher or the school administration staff. Those who agreed to participate received a large envelope containing a series of questionnaires and consent forms. All the questionnaires were clearly labeled as either the paternal or maternal version and were printed in different colors. The consent form informed the fathers and mothers to complete their questionnaires independently. Upon completion, the parents returned the questionnaires to their children’s teachers, and the schools sent the questionnaires back to the researchers once all the questionnaires were collected from the participating families. All procedures were approved by the Institutional Review Board of the sponsoring university.

### Measures

#### The Background Information Questionnaire

To assess demographic information, we developed the Background Information Questionnaire. This tool collected the following information: (1) the child’s gender, age, age when he or she was identified as having ASD, and whether the child with ASD was the only child, (2) the parents’ age, occupation, income, and educational background, and (3) the parents’ satisfaction with their marriage, self-evaluation of their status as parents, and their relationship with the child and the child’s grandparents. Additionally, the parents reported their child’s academic performance and daily living skills on a 4-point Likert scale (1 = excellent, 2 = good, 3 = moderate, 4 = poor). The children’s academic performance was included as a control variable because academic achievement is particularly emphasized in mainland China given longstanding cultural values regarding academics (Hesketh and Ding 2005). Therefore, parenting aspects of interest in the current study might be confounded with academic performance.

#### Difficulties in Emotion Regulation Scale (DERS; Gratz and Roemer 2004)

Parental emotion regulation was measured by the DERS. This 36-item self-report scale used a 5-point Likert-type response scale ranging from 1 (almost never) to 5 (almost always), with higher total scores representing greater difficulties with emotion regulation. The DERS provides a comprehensive assessment of parents’ overall emotion regulation difficulties as well as difficulties in six specific dimensions, including accepting emotional responses (e.g., “When I am upset, I become angry with myself for feeling that way”), impulse control (e.g., “When I am upset, I feel out of control”), lack of emotional awareness (e.g., “I pay attention to how I feel”, reverse scored), lack of emotional clarity (e.g., “I have no idea how I am feeling”), accessing emotion regulation strategies (e.g., “When I’m upset, I believe that I will end up feeling very depressed”), and engaging in goal-directed behavior when emotionally aroused (e.g., “When I’m upset, I have difficulty concentrating”). The current study only examined parents’ overall emotion regulation difficulties. The Chinese version of the DERS has demonstrated good psychometric properties (Li et al. 2018). The internal consistency reliability of the DERS in this study was good, with Cronbach’s αs of .87 for the paternal reports and .91 for the maternal reports.

#### The Parenting Stress Inventory-Short Form (PSI-SF; Abidin 1995)

The Chinese version of the PSI-SF was used to assess the parenting stress of parents with children with ASD. The PSI-SF is a 36-item self-report scale with three subscales: parental distress (e.g., “I feel trapped by my responsibilities as a parent”), difficult child (e.g., “My child seems to cry or fuss more than most children”), and dysfunctional parent–child interaction (e.g., “My child rarely does things for me that makes me feel good”). A 1- to 5-point Likert scale format (1 = strongly agree, 5 = strongly disagree) was used, with higher scores on the total scale representing a higher degree of parenting stress. The Chinese version of the PSI-SF has demonstrated good reliability and validity (Pearson and Chan 1993), and in this study, the internal consistency of the Chinese version was .92 for the fathers and .90 for the mothers.

#### The Parenting Bonding Instrument (PBI; Parker et al. 1979)

The Chinese version of the PBI was used to measure the parenting behaviors of the parents with children with ASD. The PBI consists of 23 items, with each item rated on a 4-point Likert scale (0 = very unlike, 3 = very like). The items ask parents to report their behaviors toward their children and are grouped into two domains: care (e.g., “I speak to my child in a warm and friendly voice”) and overprotection (e.g., “I like my child to make his/her own decisions”). Optimal parental bonding behaviors were assessed using the sum of the parental care items and the reverse-scored parental overprotection items (Cohen and Murphy, Common Cold Project). The higher the optimal score, the more likely the parents’ behaviors are to promote bonding with their children and indicate adaptive parenting. The Chinese PBI has demonstrated good reliability in previous studies with families from similar cultural backgrounds (e.g., Yan et al. 2015). In this study, the internal consistency was .81 for the fathers and .91 for the mothers for optimal bonding, .81 for the fathers and .76 for the mothers for care, and .66 for the fathers and .71 for the mothers for overprotection.

#### Missing Data

The rates of missing data on the DERS were 10.4% for the mothers and 13.7% for the fathers. The percentage of missing cases on the PSI was 10.9% for mothers and 17.5% for fathers. For the care domain of the PBI, the rates of missing data were 1.9% for mothers and 5.2% for fathers. For the overprotection domain of the PBI, the missing data rates were 6.2% for mothers and 9.5% for fathers. The missing data rates were 7.6% on maternal optimal bonding and 12.3% on paternal optimal bonding. Data were missing completely at random (Little’s MCAR *χ*^2^ = 76.70, *p* = 1.00), and missing data were handled using the full-information maximum likelihood in Mplus 7.0 (Muthén and Muthén 1998–2012).

### Data Analysis

**Fig. 1:**
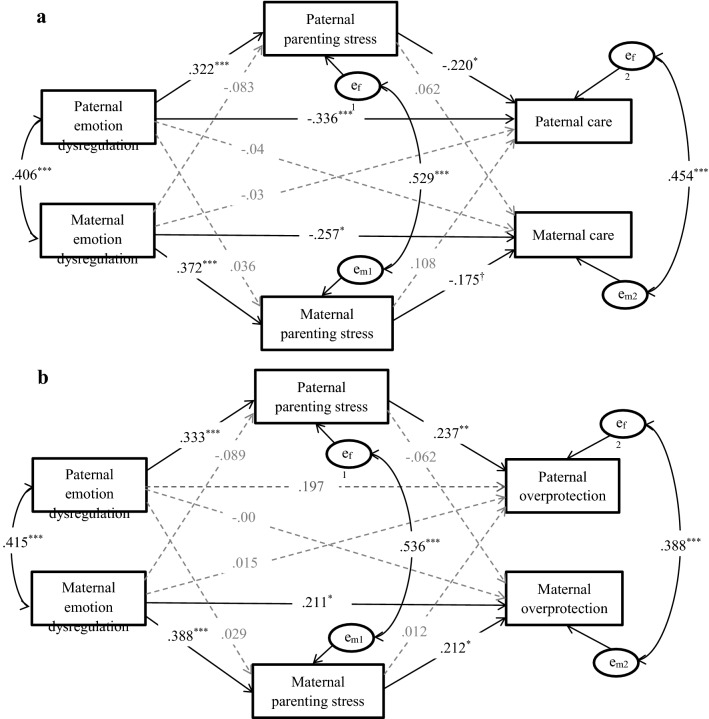
Actor–partner interdependence mediation model predicting parental care (**a**) and overprotection (**b**) from emotion dysregulation and parenting stress. *Note N* = 211. Child’s daily living skills and academic performance are controlled for parental parenting stress. The only child or not and child’s academic performance are controlled for parental care. Child’s daily living skills, academic performance, household income and paternal educational level are controlled for parental overprotection. All the control variables are not shown. All the coefficients are standardized estimates. Solid lines indicate significant/marginal significant paths, and grey dashed lines indicate non-significant paths. ^†^*p *<.10; *p < .05; **p < .01; ***p < .001

**Fig. 2:**
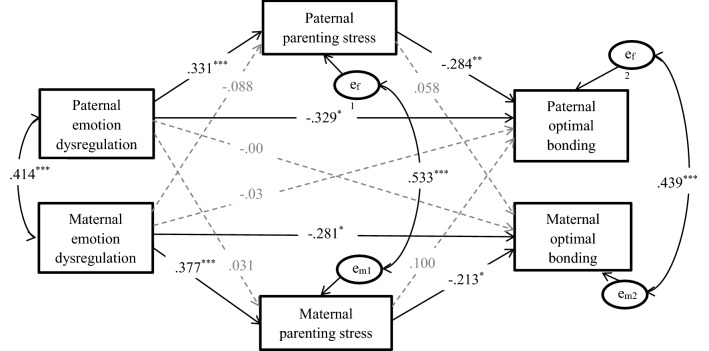
Actor–partner interdependence mediation model predicting parental optimal parenting from emotion dysregulation and parenting stress. *Note N* = 211. Child’s daily living skills and academic performance are controlled for paternal and maternal parenting stress, and child’s daily living skills, academic performance and paternal educational level are controlled for parental optimal bonding (not shown). All the coefficients are standardized estimates. Solid lines indicate significant paths, and grey dashed lines indicate non-significant paths. *p < .05; **p < .01; ***p < .001

First, the descriptive statistics and correlational analyses of the mothers and fathers were reported separately using SPSS 20.0. Second, possible group differences were analyzed based on demographic characteristics to identify potential control variables. Finally, the actor–partner interdependence mediation models (APIMeMs) in Figs. 1 and 2 were tested, as recommended by Ledermann et al. (2011), to assess the mediating role of maternal and paternal parenting stress in the paths from emotional regulation to parenting behaviors. The APIMeM allowed us to simultaneously test how one parent’s predictor variable could influence their own (actor effects) and their partners’ (partner effects) outcomes as well as the mediating mechanisms in the dyadic relationship (Ledermann and Kenny 2012). This approach has been applied in different research areas and cultures, including stress processes in Chinese culture (Xu et al. 2018). All APIMeM results were tested in Mplus 7.0 (Muthén and Muthén 1998–2012), and we utilized the bias-corrected bootstrap 95% confidence interval (CI) based on 5000 bootstrap samples.

## Results

### Preliminary Analysis

The descriptive statistics of the study variables for the mothers and fathers are shown in Table 1. The absolute kurtosis and skewness were below 2.3 and 7.0, suggesting that the total scores of mothers and fathers followed a normal distribution (Lei and Lomax 2005).

**Table 1:** Descriptive statistics and reliability of study variables

Variable	Range	Mean	SD	Kurtosis	Skewness	Cronbach’s α
Paternal measures						
DERS	52–144	79.98	15.60	0.85	0.69	.87
PSI	36–153	105.85	17.71	1.37	− 0.34	.92
PBI care	10–33	23.18	4.72	− 0.36	− 0.02	.81
PBI overprotection	3–27	15.16	4.38	0.10	− 0.12	.66
PBI optimal bonding	25–66	44.00	7.62	− 0.17	0.33	.81
Maternal measures						
DERS	43–149	82.63	18.83	0.31	0.57	.91
PSI	63–157	109.23	16.90	0.07	0.10	.90
PBI care	13–33	24.26	4.05	− 0.52	− 0.12	.76
PBI overprotection	1–28	15.35	4.64	0.37	− 0.40	.71
PBI optimal bonding	21–66	44.95		0.33	0.27	.91

*DERS* Difficulties in Emotion Regulation Scale, *PSI* Parenting Stress Inventory-Short Form, *PBI* Parenting Bonding Instrument

The Pearson’s correlations among the study variables and demographic data are shown in Table 2. The correlations of difficulties with emotion regulation, parenting stress, parental care, overprotection, and optimal parenting scores between mothers and fathers were significant, suggesting nonindependence in the dyads (see the bolded coefficients in Table 2).

**Table 2:** Bivariate correlations of study variables and demographics

	1	2	3	4	5	6	7	8	9	10	11	12	13	14	15	16	17
(1) M-DERS	–																
(2) F-DERS	.381**	–															
(3) M-PSI	.444**	.213**	–														
(4) F-PSI	.077	.303**	.520**	–													
(5) M-PBI care	− .372**	− .183*	− .278**	− .132	–												
(6) F-PBI care	− .173*	− .414**	− .080	− .313**	.440**	–											
(7) M-PBI overprotection	.291**	.105	.320**	.159*	− .450**	− .194**	–										
(8) F-PBI overprotection	.102	.278**	.219**	.330**	− .182*	− .387**	.410**	–									
(9) M-PBI optimal bonding	− .378**	− .144	− .346**	− .175*	.826**	.366**	− .875**	− .359**	–								
(10) F-PBI optimal bonding	− .163*	− .416**	− .175*	− .386**	.370**	.845**	− .364**	− .820**	.436**	–							
(11) Child age	.093	− .057	.071	.092	− .012	.098	.083	− .033	− .036	.090	–						
(12) Child gender	.069	− .103	− .013	.112	− .023	.072	− .023	.004	.021	.048	.030	–					
(13) Only child or not	.216**	.080	.075	.113	− .088	− .159*	− .044	− .087	− .025	− .052	− .073	− .048	–				
(14) Child daily living skills	.124	.112	.347**	.272**	− .100	− .112	.246**	.165*	− .204**	− .177*	− .036	− .013	.059	–			
(15) Child academic performance	.208**	.044	.312**	.253**	− .172*	− .079	.201**	.187*	− .218**	− .150*	− .013	.055	.200**	.569**	–		
(16) Household monthly income	− .219**	− .140	− .118	− .051	.126	− .005	.074	.159*	.005	− .110	− .078	.009	− .216**	− .011	− .073	–	
(17) M-educational level	.295**	.318**	.096	.047	− .087	− .083	− .060	− .058	.010	.000	.089	− .064	.276**	.004	.006	− .606**	–
(18) F-educational level	.096	.152*	− .030	− .020	.080	.002	− .172*	− .070	.161*	.043	.053	− .059	.246**	.016	− .002	− .552**	.684**

*N* = 221. Child gender: 0 = *male*, 1 = *female*. Only child or not: 1 = only child, 2 = not only child. Parental educational level: 1 = *elementary school diploma*, 6 = *master*’*s degree or above*. Household monthly income: 1 = *0*–*1999 Chinese yuan or less*, 10 = *9999 Chinese yuan or more*. The nonindependence in dyads are *bolded*

*F*/*M*-*DERS* total score of paternal/maternal Difficulties in Emotion Regulation Scale, *F*/*M*-*PSI* total score of paternal/maternal Parenting Stress Inventory-Short Form, *F*/*M*-*PBI* paternal/maternal Parenting Bonding Instrument

*p < .05, **p < .01, ***p < .001

#### Covariates

In addition, several demographic variables were significantly correlated with the study variables. As shown in Table 2, fathers of the target children who were the only child in the family reported higher paternal care scores than those of the target children who had siblings. The child’s daily living skills and academic performance were significantly positively correlated with paternal and maternal parenting stress and overprotection and were significantly negatively correlated with maternal and optimal parenting behaviors. The children’s academic performance was also significantly associated with maternal care scores. In addition, household monthly income was significantly associated with paternal overprotection, and fathers’ educational level was significantly correlated with maternal overprotection and optimal bonding.

Based on the correlational results, the children’s daily living skills and academic performance were controlled for paternal and maternal parenting stress. Only the child’s status and children’s academic performance were controlled for parental care. The children’s daily living skills, academic performance, household income, and paternal education level were controlled for parental overprotection. The children’s daily living skills, academic performance and paternal educational level were controlled for parental optimal bonding in later analyses.

#### Associations Between Emotion Regulation, Parenting Stress, and Parenting Behaviors

As shown in Table 2, the associations between parents’ emotion dysregulation and their own parenting stress were significant. Paternal emotion dysregulation was correlated with maternal perception of parenting stress, whereas maternal parenting stress was not related to paternal parenting stress. Parenting stress of parents was associated with overprotection and optimal bonding for both parents but was only related to parents’ own parental care.

### APIMeM Analyses

APIMeM analysis was conducted to examine the indirect effects of parents’ emotional dysregulation on paternal care, overprotection, and optimal parenting through both maternal and paternal parenting stress. Before the analysis, we performed the omnibus test of distinguishability to examine the differences in the means, variances, and covariances between fathers and mothers to establish a corresponding model (Kenny et al. 2006). The results suggested that the gender difference between mothers and fathers was significant for the model with parental care as the outcome variable (*χ*^2^[12] = 46.94, *p *< .001), the model with parental overprotection as the outcome variable (*χ*^2^[12] = 21.04, *p *< .050), and the model with parental optimal parenting as an outcome variable (*χ*^2^[12] = 28.77, *p *= .004). Therefore, the distinguishing APIMeM was estimated in the following analyses (as shown in Figs. 1, 2).

#### Model with Parental Care as the Outcome Variable

For the model with parents’ parental care as the outcome variable, children’s daily living skills and academic performance were controlled for paternal and maternal parenting stress, and only child status and academic performance were controlled for parental care, as mentioned above. The model fit the data, *χ*^2^(4) = 2.53, *p *= .639, *CFI *= 1.000, *TLI *= 1.041, *RMSEA *= .000, *SRMR *= .010.

As shown in Fig. 1a, parents’ difficulties with emotion regulation were directly and positively associated with their own parenting stress and negatively related to their own parental care, but they were not associated with their spouses’ parenting stress or parental care. The relations between maternal parenting stress and maternal care was marginally significant, and paternal parenting stress was significantly and negatively related to paternal care. No partner effect was found for the relations between parenting stress and parental care. Moreover, the indirect effect of parents’ emotion dysregulation on parental care through parenting stress was only found for the fathers. Specifically, the links between the fathers’ difficulties with emotion regulation and their own parental care were mediated by the fathers’ perceived parenting stress. Note that the indirect effects were only marginally significant: *β *=− .07, *SE* = .04, *p* = .058, 95% *CI* = [− 0.144, 0.002]. No other actor/partner-level indirect effect was found.

#### Model with Parental Overprotection as the Outcome Variable

For the model with parental overprotection as the outcome variable, the children’s daily living skills and academic performance were controlled for paternal and maternal parenting stress, and the children’s daily living skills, academic performance, household income and paternal educational level were controlled for parental overprotection, as mentioned. The model fit the data, *χ*^2^(4) = 4.07, *p *= .396, *CFI *= 1.000, *TLI *= .998, *RMSEA *= .009, *SRMR *= .014.

As shown in Fig. 1b, parents’ difficulties with emotion regulation were positively related to their own parenting stress but not to their spouses’ parenting stress. Maternal difficulties with emotion regulation were significantly and positively associated with maternal overprotection. However, the relations between paternal difficulties with emotion regulation and overprotection was not significant. The relations between parenting stress and parent’s own parental overprotection were significant for both fathers and mothers, such that more parenting stress was associated with more parental overprotection. No partner effect was found in the model. Further analyses suggested that the fathers’ difficulties with emotion regulation had indirect effects on their own paternal overprotection via their self-reported parenting stress, *β *=.08, *SE* = .04, *p* = .024, 95% *CI* = [0.010, 0.148]. In addition, the indirect effect of maternal emotion dysregulation on their own overprotection through maternal parenting stress was marginally significant, *β *=.08, *SE* = .04, *p* = .058, 95% *CI* = [− 0.003, 0.167]. None of the other actor/partner-level indirect effects was significant.

#### Model with Optimal Parenting as the Outcome Variable

When optimal parenting was investigated as the outcome variable, the children’s daily living skills and academic performance were controlled for paternal and maternal parenting stress; children’s daily living skills, academic performance, and paternal educational level were controlled for this model based on correlational results. The model showed a good fit, *χ*^2^(2) = 2.07, *p *= .356, *CFI *= 1.000, *TLI *= .996, *RMSEA *= .013, *SRMR *= .014.

As shown in Fig. 2, we found a significant actor effect but no partner effect. Specifically, for these Chinese parents, (1) the fathers’ and mothers’ difficulties with emotional regulation were significantly and positively associated with their own parenting stress: parents with more difficulties with emotional regulation had higher levels of parenting stress, (2) fathers’ and mothers’ parenting stress were significantly and negatively related to their own optimal parenting: parents with higher levels of parenting stress had lower parenting scores, and (3) difficulties with emotion regulation had a significant and negative direct correlation with optimal parenting, even after controlling for parenting stress. Specifically, after the effect of parenting stress was ruled out, parents who had more difficulties with emotion regulation reported lower optimal parenting scores.

The mediation analyses (see Table 3) suggested that for both mothers and fathers, difficulties with emotion regulation were significantly associated with their own parenting behaviors through their own parenting stress (for mothers, *β *=− .08, *SE* = .04, *p* = .043, 95% *CI* = [− 0.158, − 0.003]; for fathers, *β *= − .09, *SE* = .04, *p* = .030, 95% *CI* = [− 0.179, − 0.009]) but not through their spouses’ parenting stress.

**Table 3:** Total, direct, and indirect actor and partner effects for actor–partner interdependence mediation model with paternal and maternal emotion dysregulation as predictors, parenting stress as mediators, and parental optimal bonding as outcome variables

Effect	b	β	p (two-tailed)	95% CI
Paternal DERS → paternal optimal bonding				
Total effect	**−** **0.199**	**−** **.420**	**.000**	**[−** **0.564, −** **0.275]**
Total indirect effect	**−** **0.043**	**−** **.091**	**.025**	**[−** **0.170, −** **0.011]**
Paternal DERS → paternal PSI → paternal optimal bonding	**−** **0.045**	**−** **.094**	**.030**	**[−** **0.179, −** **0.009]**
Paternal DERS → maternal PSI → paternal optimal bonding	0.001	.003	.771	[− 0.018, 0.024]
Direct effect	**−** **0.156**	**−** **.329**	**.000**	**[−** **0.503, −** **0.155]**
Maternal DERS → maternal optimal bonding				
Total effect	**−** **0.143**	**−** **.366**	**.000**	**[−** **0.497, −** **0.236]**
Total indirect effect	− 0.033	− .085	.050	[− 0.171, 0.000]
Maternal DERS → maternal PSI → maternal optimal bonding	**−** **0.031**	**−** **.080**	**.043**	**[−** **0.158, −** **0.003]**
Maternal DERS → paternal PSI → maternal optimal bonding	− 0.002	− .005	.673	[− 0.029, 0.019]
Direct effect	**−** **0.110**	**−** **.281**	**.000**	**[−** **0.439, −** **0.123]**
Maternal DERS → paternal optimal bonding				
Total effect	0.012	.029	.716	[− 0.127, 0.185]
Total indirect effect	0.025	.063	.181	[− 0.029, 0.155]
Maternal DERS → maternal PSI → paternal optimal bonding	0.015	.038	.362	[− 0.044, 0.119]
Maternal DERS → paternal PSI → paternal optimal bonding	0.010	.025	.340	[− 0.026, 0.076]
Direct effect	− 0.014	− .034	.713	[− 0.214, 0.146]
Paternal DERS → maternal optimal bonding				
Total effect	0.004	.010	.898	[− 0.137, 0.157]
Total indirect effect	0.006	.013	.731	[− 0.059, 0.084]
Paternal DERS → paternal PSI → maternal optimal bonding	0.009	.019	.555	[− 0.045, 0.083]
Paternal DERS → maternal PSI → maternal optimal bonding	− 0.003	− .007	.677	[− 0.038, 0.025]
Direct effect	− 0.001	− .003	.973	[− 0.170, 0.164]

*N* = 192. Results in bold indicate significant coefficients. Child’s daily living skills and academic performance are controlled for paternal and maternal parenting stress, and child’s daily living skills, academic performance and paternal educational level are controlled for paternal and maternal optimal bonding

*DERS* total score of Difficulties in Emotion Regulation Scale, *PSI* total score of Parenting Stress Inventory-Short Form, *CI* confidence interval

## Discussion

The goal of the current study was to elucidate the relationships among Chinese parents’ emotion regulation, parenting stress, and parenting behaviors in families of children with ASD while considering the effects of the dynamic interactions between fathers and mothers. This study is among the first to utilize dyadic analysis (i.e., actor partner interdependence mediation model) to investigate the potential spillover and crossover effects within families of children with ASD. The findings of the current study improve our understanding of how emotional factors contribute to parenting stress and how emotional factors are associated with the behaviors of fathers and mothers when parenting their children with ASD. These results have important implications for potential targeted intervention programs for parents of children with ASD, that the emotion regulation training programs for parents may decrease perceived parenting stress and thus promote more adaptive parenting behaviors toward children with ASD.

First, the correlational results suggested nonindependence between fathers and mothers. Specifically, significant correlations were found between fathers and mothers for emotion dysregulation, parenting stress and parenting behaviors (i.e., care, overprotection, and optimal bonding). Therefore, it is important to assess fathers’ and mothers’ influences in the same model to understand the potential spillover and crossover effects in the families of children with ASD. In addition, the correlational results suggested that Chinese mothers’ heightened levels of difficulties with emotion regulation were associated with an increase of their own parenting stress but not that of their spouses, while when fathers reported more difficulties with emotion regulation, both parents’ reported higher perceived stress when providing care to their children with ASD. We also found significant correlations between Chinese parenting stress and both parents’ care, overprotection and optimal parenting behaviors. The only exception was the link between maternal stress and paternal care; specifically, mothers’ parenting stress was not significantly associated with their spouses’ care behaviors. These correlational results suggest that fathers and mothers may have differential roles within family systems when co-parenting children with ASD (Cridland et al. 2014): compared to fathers, Chinese and that mothers may be more subject to spillover and crossover effects within family systems when facing emotional challenges.

The APIMeMs were then applied to test our main hypotheses. We first hypothesized that a higher level of emotion dysregulation would be directly associated with heightened parenting stress, increased negative parenting behaviors, and decreased positive parenting behaviors for both parents in Chinese families of children with ASD. Additionally, we hypothesized that both fathers’ and mothers’ perceived stress from being a parent would be associated with their own and their partners’ parenting behaviors. The actor effects of these links, but not the partner effects, were supported by our results.

Specifically, consistent with the correlational results, Chinese parents with greater emotion regulation difficulties self-reported more parenting stress, and parents with higher levels of parenting stress self-reported more parental overprotection/control, less care/affection, and less optimal bonding with their children with ASD, even after controlling for the spouses’ emotion dysregulation and parenting stress. This finding provided empirical evidence to theoretical propositions regarding the relationships between parental emotional well-being and parenting behaviors in families of children with ASD (Dix 1991; Adam et al. 2004). Note that the direct effects of parental emotion dysregulation on self-reported parenting behaviors remained significant after controlling for spouses’ emotion dysregulation and both parents’ parenting stress. These results suggest that other possible mediators, such as marital relationships and co-parenting, may exist under these mechanisms (e.g., Katz and Gottman 1996).

We then tested our mediational hypotheses regarding the dyadic interactions among fathers’ and mothers’ emotion regulation, parenting stress, and parenting behaviors within the family system of children with ASD. The results mostly supported our hypotheses on actor, but not partner levels, suggesting the existence of a spillover rather than crossover effect within families of children with ASD. Specifically, parents who showed more difficulties with emotion regulation were more likely to perceive parenting stress, which in turn was associated with fewer optimal bonding behaviors, more overprotection, and less care toward their child. However, there was one exception: maternal parenting stress did not significantly mediate the links between maternal emotion dysregulation and maternal care.

Specifically, it seemed that for both the mothers and fathers of children with ASD in Chinese culture, difficulties coping with daily emotional challenges might be linked to their elevated levels of perceived stress when caring for their children with ASD. This heightened stress perception, in turn, seemed to relate to their use of optimal bonding behaviors with their children. Additionally, these pathways remained significant even after other factors, such as children’s daily living skills, household income, number of children in the family and spouses’ parenting stress and emotion dysregulation, were considered. Note that although we found only a marginal mediating effect of self-reported parenting stress on the link between Chinese parents’ emotion dysregulation and overprotection for mothers and on the link between emotion dysregulation and care for fathers, the pattern of results is still meaningful and consistent with our predictions.

These actor-level results were consistent with the theoretical proposition that the emotions of the individuals (e.g., parents’ emotions) within the family systems could directly influence those individuals’ relationship with their child (e.g., parent–child relationship; Cox and Paley 2003). This is one of few studies to use family systems approach to examine the family dynamics in families of children with developmental disabilities, despite the continuous call to adopt family systems approach as a guiding framework for family-focused ASD research (Cridland et al. 2014). These results indicate the importance of providing emotional support and training in emotion regulation strategies to both parents and to include both fathers and mothers as target primary caregiver in clinical support services. In China, mothers of children with ASD have long been the service targets in clinics and agencies that aim to provide interventions for children with ASD in home settings, and fathers have always been ignored (China Association of Persons with Psychiatric Disability and Their Relatives 2014). Systematic training on emotion regulation for both mothers and fathers should be provided in clinics and agencies to empower parents with emotion coping strategies. Furthermore, the provision of in-home family support programs for both parents by local government agencies in charge of affairs for children with autism (i.e., local association for people with disabilities) will help mothers and fathers respond to parenting pressure and challenges positively by teaching them how to recognize emotional pressure and strength, act as role models, promote resilience, and build competence (Wang and Hu 2014).

The partner-level pathways, however, were not significant for either positive or negative parenting behaviors. Although the crossover process of family systems theory (Erel and Burman 1995) suggests that parents’ emotion regulation might be both associated with their own parenting behaviors through its impact on perceived parenting stress and linked to their spouses’ parenting behaviors through their impact on the spouses’ stress perception, we did not find such crossover effects in our sample of Chinese families of children with ASD.

On the surface, these results imply that one partner’ emotion dysregulation and parenting stress may not contribute to the other partner’s parenting behaviors beyond the individual’s own emotion dysregulation and parenting stress in this specific group (i.e., Chinese parents of children with ASD). One possible explanation is that unlike in other groups of children (e.g., typically developing children), the distinct challenges associated with ASD might lead these Chinese parents to be overly occupied with dealing with their children’s core symptoms and mediating the children’s social interactions (e.g., Heiman and Berger 2008). This focus often means that parents must constantly cope with their children’s lack of spontaneity, sudden mood changes, and inflexible daily routines (Latefa and Ahmad 2015; Pakenham et al. 2004). Such heightened demands might deplete parents’ opportunities to become highly involved in interactions with their co-parents. Another possible explanation is that mothers and fathers have clear-cut, distinct roles in daily family life, especially within the traditional Chinese cultural background. Specifically, fathers may be responsible for supporting, providing for, and protecting his family by paying for all family expenses and the costs of interventions and therapy for the child with ASD, while mothers usually stay home to take care of the child with ASD (China Association of Persons with Psychiatric Disability and Their Relatives 2014). Additionally, compared with families within the Western culture, emotional interactions between Chinese mothers and fathers are introverted and implicit (Ma and Lai 2016). Such family values and role divisions might impede spousal communication, bonding, and closeness.

Although no partner effects were found in our models, other potential partner-level pathways could still existing. For example, emotion dysregulation and parenting stress were correlated within couples, so it is possible that one parent’s emotion dysregulation and parenting stress influence his/her partner’s parenting behaviors through the bidirectional exchanges between fathers and mothers on emotion dysregulation and parenting stress. Previous work has supported the interconnectedness of daily emotions in marital relationships (Schoebi 2008) and the correlations between couples’ self-reported parenting stress in families of children with ASD (Pisula and Porębowicz-Dörsmann 2017); thus, it is possible that the participants’ parenting stress and behaviors were influenced by their partners’ emotion dysregulation through the coregulation processes within couples. Of course, the cross-sectional design of our study limited our ability to make any statement regarding time order or causality.

Although this study expands the parenting literature on ASD from a dyadic perspective, multiple limitations exist and warrant caution for interpretation. First, the present study was limited by its reliance on paternal and maternal self-reports. At present, the findings should be interpreted with caution as the study was subject to potential biases, such as social desirability bias and response bias. For example, the major study variables, such as parenting emotion regulation and parenting behaviors, and the covariates, such as child academic performance and daily living skills, were assessed only by the parents and may have been biased by parents’ tendency to give socially acceptable answers, even if they are not true. Future studies should consider assessing these variables through multiple approaches (e.g., behavioral observation, psychophysiological assessment, teachers’ reports) to overcome the issues with social desirability and response bias and create a more comprehensive picture.

Second, other covariates, such as child intellectual abilities, should be considered. Unfortunately, we were not able to obtain the children’s IQ information. Although we included child academic performance, which is strongly correlated with intellectual abilities in children with ASD (e.g., Venter et al. 1992), other studies have shown significant discrepancies between intellectual abilities and academic performance (Estes et al. 2011). Thus, future studies that include other important covariates (e.g., child IQ) and assess covariates using more sophisticated instruments and multiple perspectives (e.g., teachers’ report, behavioral observation) are warranted. Third, the cross-sectional design of the current study limited our abilities to test causal relations or determine the directionality of the associations among examined variables. Future longitudinal studies are strongly encouraged to investigate the causality and relationships among these parenting constructs and to measure changes in these constructs over time.

Additionally, the optimal parenting subscale created in the current study was consistent with studies with typically developing children and children with other types of psychopathology (e.g., ADHD, anxiety disorder); however, whether overprotection can always be considered a negative parenting characteristic should be considered with caution. Future studies are strongly encouraged to improve our understanding of this parenting behavior in families of children with ASD. Lastly, although our study was not designed in a cross-cultural manner, this emerging evidence suggests that future studies should include measures of culturally relevant constructs (e.g., parents’ emotion regulation) during their investigations.

In summary, our results support the existence of spillover effects (but not crossover effects) in the relationships among parental emotion regulation, parenting stress and parenting behaviors. The present study contributes to the literature in several important ways. First, our study is among the first to investigate the mechanisms of the links between emotion regulation and parenting behaviors by including both parents’ parenting stress as mediators. Second, it examined these relationships using dyadic analysis, enabling an examination of whether potential family processes (i.e., spillover and crossover processes) exist under stricter control. Third, our findings further indicate the need to provide family-centered support to both mothers and fathers to enhance their roles in parenting children with ASD in China (Hu et al. 2015). Support for parents might include, but should not be limited to, empowerment strategies, parent–child relationship-building skills, coping skills for working with challenging behaviors, and spousal emotional assistance strategies. In addition to providing and tailoring support and services to parents, education programs for addressing and enhancing daily living skills of school-aged children with ASD should be prioritized because daily living skills were significantly correlated with parenting stress. Daily living skills programs should be integrated into current teaching programs at special education schools for children with ASD.
